# Minimally Invasive Surgical Excision of 101 Lipomas in Familial Multiple Lipomatosis: A Case Report

**DOI:** 10.7759/cureus.88720

**Published:** 2025-07-25

**Authors:** Kieron Young, Dardan Popova, Tonderai Mutsago, Caroline Payne

**Affiliations:** 1 Plastic and Reconstructive Surgery, The Royal London Hospital, London, GBR

**Keywords:** complete excision, familial multiple lipomatosis, lipoma, lipoma excision, minimally invasive surgeries, minimally invasive surgical technique (mist), minimally invasive surgery

## Abstract

Familial multiple lipomatosis (FML) is a rare autosomal dominant condition characterised by multiple subcutaneous lipomas affecting the trunk and extremities. Although benign, the aesthetic and functional burden of multiple lipomas often prompts patients to seek treatment. We present a 57-year-old male with a 15-year history of FML who underwent minimally invasive surgical excision of 101 forearm lipomas through 34 strategically placed incisions. The patient recovered well, returned to manual work within one week, and reported high cosmetic satisfaction. At the two-year follow-up, only three asymptomatic recurrences were noted on the left forearm. This case highlights a minimally invasive technique that optimises cosmetic outcomes while maintaining effective lesion clearance in patients with multiple lipomas.

## Introduction

Familial multiple lipomatosis (FML) is a rare benign condition characterised by a diffuse distribution of subcutaneous, encapsulated, non-tender lipomas across the trunk and extremities while sparing the head and neck [1].​​ It is inherited in an autosomal dominant manner, typically affecting men and women in the third decade of their life, with an estimated prevalence of 1 in 50,000 [2,3].​ While the underlying genetic aetiology is yet to be identified, genetic abnormalities in HMGA2 (High Mobility Group At-Hook 2) and PALB2 (Partner And Localizer of BRCA2) are implicated in its development [2].

Having multiple lipomas can significantly impact a patient’s appearance and overall quality of life, often making treatment necessary. The most common treatment options are surgical excision and liposuction [3,4].​ While excision is effective, it typically leaves scarring over the site where each lipoma has been excised, which can be particularly undesirable when multiple lipomas are present. In contrast, liposuction is less invasive but can fail to completely remove the lipoma [4].​

We report a case of FML in a 57-year-old male who presented with multiple subcutaneous lipomas across both forearms. We describe the minimally invasive approach taken to excise 101 of these lipomas. This technique focused on the removal of multiple lipomas through a single incision, aiming to minimise scarring while ensuring effective clearance.

## Case presentation

**Figure 1:**
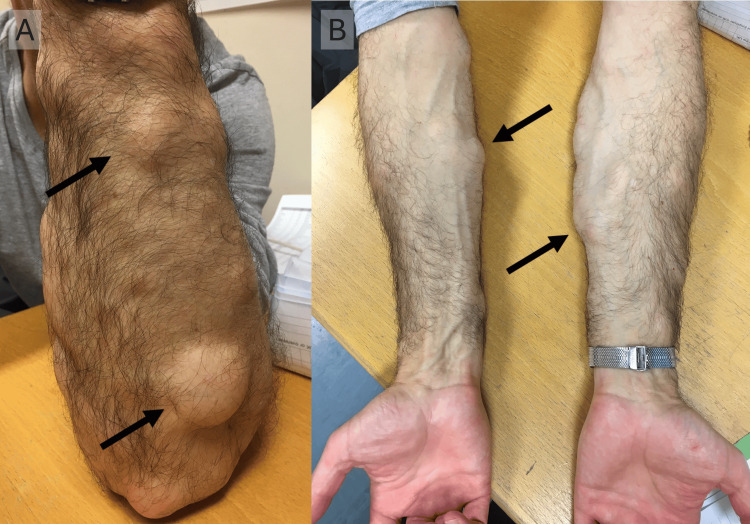
Pre-operative photographs showing the patient’s (A) masses on the posterior aspect of the left forearm and (B) the masses on the anterior aspect of both forearms Arrows point to the subcutaneous masses in the forearms.

A 57-year-old Caucasian male, with a 15-year history of FML, presented with multiple, non-tender, subcutaneous lumps on the anterior and posterior aspects of both upper limbs, particularly on the forearms (Figures 1A, 1B). Additional lipomas were present on the abdomen and back but were reported as less bothersome. Examination revealed numerous mobile and discrete rubbery masses ranging from 1 to 8 centimetres. No visible changes were observed on the overlying skin. Clinical palpation alone was used to assess the lesions pre-operatively.

The patient was eager to remove the lumps on both upper limbs, especially those distributed across his forearms, which were easily visible and often commented on by others. It was agreed that all palpable lipomas bilaterally on the upper limbs would be surgically excised for cosmetic purposes. Written informed consent was obtained after a detailed discussion of the nature of the procedure, associated risks, and expected outcomes.

**Figure 2:**
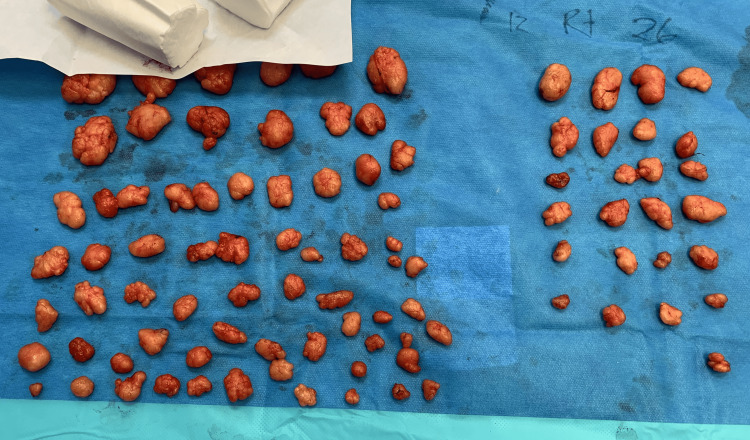
Intraoperative photograph showing the lipomas removed

The procedure was performed under general anaesthesia with a local anaesthetic infiltration of 1% xylocaine with 1:200,000 adrenaline, diluted in normal saline solution (0.9% NaCl), without arm tourniquets. Twenty-two incisions on the left arm and 12 incisions on the right arm were made to remove 69 and 32 lipomas, respectively. Lesions ranged from 1.0x0.7cm to 7.2x6.0cm in size (Figure 2). The incisions were strategically placed between multiple nearby lipomas so that, with a small amount of undermining combined with sharp and blunt dissection, multiple adjacent lipomas could be accessed and safely removed. When planning the incisions, care was taken to avoid compromise to the vascularity of the skin flaps. Histological analysis performed on one specimen per arm revealed well-encapsulated fibrofatty tissue. Monocryl subcuticular suture was used for skin closure, so no suture removal was required. The surgical procedure lasted 120 minutes, and the patient was discharged from the hospital the same day. There were no intra- or post-operative complications.

**Figure 3:**
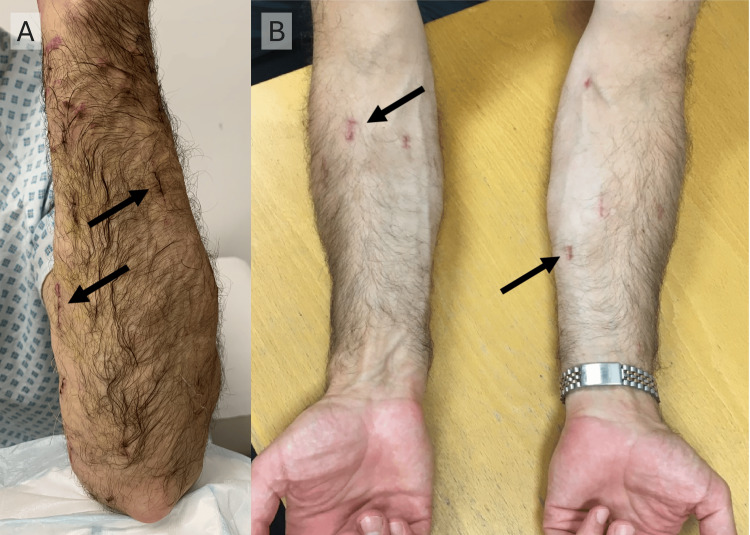
Photographs showing the patient’s (A) posterior aspect of the left forearm one week postoperatively and (B) both forearms two months postoperatively Arrows point to incision sites on the forearms.

He recovered fully within one week and promptly returned to manual work. There was only minimal bruising in the first post-operative week, and the patient reported satisfaction with the final outcome (Figures 3A, 3B). Two years later, the patient reported the recurrence of three new lumps exclusively on the left forearm. However, these were not bothersome to the patient at the time of follow-up.

## Discussion

Urgent intervention for the surgical excision of multiple lipomas in FML is not required owing to their pathologically benign nature and lack of malignant transformation. However, the treatment of FML must be considered in terms of the functional and cosmetic implications caused by the many subcutaneous masses. Abdominal lipomas have been described to restrict bending, while lipomas overlaying tendons in the upper limbs can cause discomfort upon movement [5,6].​ Should lipomas grow to a significant size, they can restrict daily activities by limiting motion over flexure sites. Aesthetically sensitive areas, including the upper and lower limbs, are also widely affected by FML [4,7-9]. The multiple lipomas cause disfigurement, ultimately encouraging patients to seek treatment. Fortunately, FML does not arise on the head and neck, where functional and cosmetic impairment, as well as surgical treatment, would likely be more complicated.

The surgical removal of one or multiple lipomas is usually done through a skin incision directly overlying the lump, from which the encapsulated lipomatous lesion is then ‘shelled out’ in toto [5].​ Where patients present with multiple lipomas in FML, this surgical technique can produce a large number of incisions, resulting in excessive scarring and post-operative pain. More cosmetically desirable outcomes can be achieved with a minimally invasive approach, as described by our case.

The excision of multiple non-fragmented lipomas through a single incision with digital manipulation enabled 69 and 32 lipomas to be removed from only 22 and 12 incisions, respectively. This approach was also employed by Ronan et al., who reported similar excellent cosmetic outcomes [3].​ Our patient also received a follow-up two years later, who reported a recurrence of three lipomas exclusively in the left forearm. Ronan et al. did not provide any further follow-up [3].​ To date, there are no longitudinal studies or detailed data on the prognosis of FML and its therapeutic management [2].​ Considering the absence of malignant potential in FML, routine intensive surveillance is not warranted [10].​ However, a patient-centred approach to follow-up remains appropriate, particularly in cases where recurrent or new lipomas cause functional impairment or impact quality of life. While no formal patient-reported outcome measures (PROMs) were utilised in our case, the patient expressed a high level of satisfaction with both the functional and cosmetic outcomes. Future case series or prospective studies incorporating validated PROMs would help capture a broader understanding of patient-centred benefits. 

Other approaches have also been considered for cosmesis, including the use of liposuction. However, there may be notable disadvantages when compared to our minimally invasive approach. While liposuction alone further minimises the number of incisions required and allows for strategic scar placement in more desirable locations, there is, however, a potential for higher rates of recurrence [4]. Fragmentation of lipomas during liposuction may result in their incomplete removal, whereas an excisional approach allows for the tumour to be removed in its entirety [3].​ Successfully excised well-encapsulated lipomas consequently report a recurrence rate of only 1-2% [11].​ Nevertheless, it is important to note that the quoted recurrence rate describes subcutaneous lipomas generally and not those in patients with FML.

Nerve injury is a recognised risk in any surgical excision, particularly in areas of dense neurovascular anatomy or when large numbers of lesions are removed. In our case, all lesions were located superficial to the fascia, and therefore, the risk of major nerve injury was minimal. Although the potential for injury to small superficial cutaneous nerves exists during dissection, our patient did not report any significant paraesthesia or sensory disturbance in the postoperative period, though this was not formally assessed. Traditional excision techniques using multiple incisions result in a large cumulative dissection area. This increases the chance of encountering and possibly injuring small superficial nerves, potentially leading to temporary or permanent paraesthesia, numbness, or neuropathic pain.

## Conclusions

This case highlights a successful and cosmetically favourable approach to managing familial multiple lipomatosis using a minimally invasive technique. By strategically planning incisions to allow access to multiple adjacent lipomas, many lesions were excised with minimal scarring and excellent patient satisfaction. While recurrence may still occur, as seen in our patient, further management with a pragmatic approach, assessing functional impact and the impact on quality of life, should be undertaken. A systematic review of the existing literature on FML, focusing on clinical presentations, treatment options, and recurrence rates, would better place our surgical technique as a viable intervention amongst the varying treatment modalities available. We acknowledge that our single case provides only limited data, and long-term follow-up with further comparative studies are essential to clarify recurrence rates and patient outcomes across the approaches. 
